# Complications Following Endoscopic Retrograde Cholangiopancreatography: Minimal Invasive Surgical Recommendations

**DOI:** 10.1371/journal.pone.0113073

**Published:** 2014-11-26

**Authors:** Bora Koc, Huseyin Yuce Bircan, Gokhan Adas, Ozgur Kemik, Adem Akcakaya, Alpaslan Yavuz, Servet Karahan

**Affiliations:** 1 Department of Surgery, Okmeydani Training and Research Hospital, Istanbul, Turkey; 2 Department of Surgery, Baskent University Faculty of Medicine, Istanbul Research Hospital, Istanbul, Turkey; 3 Department of Surgery, Yuzuncu Yil University Faculty of Medicine, Van, Turkey; 4 Department of Radiology, Yuzuncu Yil University Faculty of Medicine, Van, Turkey; The University of Hong Kong, Hong Kong

## Abstract

**Background:**

ERCP has a complication rate ranging between 4% and 16% such as post-ERCP pancreatitis, hemorrhage, cholangitis and perforation. Perforation rate was reported as 0.08% to 1% and mortality rate up to 1.5%. Besides, injury related death rate is 16% to 18%. In this study we aimed to present a retrospective review of our experience with post ERCP-related perforations, reveal the type of injuries and management recommendations with the minimally invasive approaches.

**Methods:**

Medical records of 28 patients treated for ERCP-related perforations in Okmeydani Training and Research Hospital between March 2007 and March 2013 were reviewed retrospectively. Patient age, gender, comorbidities, ERCP indication, ERCP findings and details were analyzed. All previous and current clinical history, laboratory and radiological findings were used to assess the evaluation of perforations.

**Results:**

Between March 2007 and March 2013, 2972 ERCPs were performed, 28 (0.94%) of which resulted in ERCP-related perforations. 10 of them were men (35.8%) and 18 women (64.2%). Mean age was 53.36±14.12 years with a range of 28 to 78 years. 14 (50%) patients were managed conservatively, while 14 (50%) were managed surgically. In 6 patients, laparoscopic exploration was performed due to the failure of non-surgical management. In 6 of the patients that ERCP-related perforation was suspected during or within 2 hours after ERCP, underwent to surgery primarily. There were two mortalities. The mean length of hospitalization stay was 10.46±2.83 days. The overall mortality rate was 7.1%.

**Conclusion:**

Successful management of ERCP-related perforation requires immediate diagnosis and early decision to decide whether to manage conservatively or surgically. Although traditionally conventional surgical approaches have been suggested for the treatment of perforations, laparoscopic techniques may be used in well-chosen cases especially in type II, III and IV perforations.

## Introduction

Endoscopic Retrograde Cholangiopancreatography (ERCP) which is an important diagnostic and therapeutic modality for disorders of biliary tree and pancreas, has evolved over the decades, since first introduced in 1968 by McCune et al [1]. ERCP has a complication rate ranging between 4% and 16% such as post-ERCP pancreatitis, hemorrhage, cholangitis and perforation. Perforation rate was reported as 0.08% to 1% and mortality rate up to 1.5% [2]–[6]. Besides, injury related death rate is 16% to 18% [7], [8].

Since the first endoscopic pancreatogram was obtained in 1968 and biliary sphincterotomy was first described in 1974, papillotomy for the management of choledocholithiasis have been widely used and in subsequent years, numerous endoscopic techniques evolved to address pancreaticobiliary disease [1], [9]. As the indications for ERCP have increased, a greater focus on recognizing and preventing complications has emerged [10]. ERCP has a complication rate ranging from 4% to 16% including asymptomatic hyperamylasemia, cardiopulmonary depression, hypoxia, aspiration, intestinal perforation, bleeding, cholangitis, adverse medication reactions, sepsis, acute pancreatitis and death. ERCP-related perforation is a rare but serious complication. The incidences of perforation reported by recent series were ranged from 0.3% to 1.3% [4], [6], [11]–[14].

The most important point in the management of ERCP-related perforations is the definition of the injury type. However, the unusual and unexpected complications are difficult to manage. The treatment of perforations varies from conservative management to urgent surgery according to the injury type and time of diagnosis. Majority of cases are retroperitoneal duodenal perforations usually due to papillotomy, whereas intraperitoneal perforations are less common and caused by the endoscope itself [15]. There has not been a consensus on management guidelines of ERCP related perforations, because of its low rate. There has been few case series in the literature that recommend different therapeutic modalities for ERCP-related perforation. Extensive drainage, repair with omental patch, pyloric exclusion, gastrojejunostomi, T-tube with or without cholecystectomy are surgical interventions that are used for the treatment of ERCP-related perforations [16]–[18]. Percutaneous drainage technique are generally used in the patients who managed conservatively.

Most recent studies indicate that, carefully selected patients may recover uneventfully with conservative management alone, while in the past, many authors advocated early surgical management for ERCP-related perforations [11], [19]. Many treatment guidelines have been proposed, but unfortunately there is still no consensus on it. Advances in laparoscopy and endoscopy led up to treat these unfortunate patients with minimal invasive techniques. In this study we want to present a retrospective review of our experience with post ERCP-related perforations, reveal the type of injuries and management recommendations with the minimally invasive approaches.

## Materials and Methods

Medical records of 28 patients treated for ERCP-related perforations in Okmeydani Training and Research Hospital between March 2007 and March 2013 were reviewed retrospectively. This study was approved by the institutional review board at our institution (Ethic Committee of Okmeydani Training and Research Hospital, Istanbul, Turkey) and informed written consent was obtained from all of the reviewed subjects for their clinical records to be used in this study.

Patient age, gender, comorbidities, ERCP indication, ERCP findings and details were analyzed. All previous and current clinical history, laboratory and radiological findings were used to assess the evaluation of perforations. Computerized tomography was planned on the onset of symptoms and repeated according the severity of the symptoms. Time between diagnosis of perforation and surgery (when used), the type of the operative intervention, the length of hospital stay, the complication rate and the ultimate patient outcome were also studied. The perforations were classified according to the site of perforation using the classification previously defined by Stapfer [11] (**Table 1**). Institutional ethic committee approved the evaluation of human subjects and the reporting of this study.

**Table 1:** Classification of ERCP-Related Perforations [19].

Type	Definition
**1**	Lateral or medial duodenal wall perforation (endoscope related)
**2**	Periampullary perforations (sphincterotomy related)
**3**	Ductal and duodenal perforations due to endoscopic instruments (not guide-wire)
**4**	Presence of retroperitoneal air due to guide-wire

According to the management policy of our institution for ERCP-related perforations; extensive contrast extravasation on ERCP/CT, extraperitoneal or intraperitoneal fluid collection on CT with unsolved problem and severe peritonitis, duodenum lateral wall or jejunal injury and problem remaining unsolved with endoscopic procedure (retained hardware or biliary stone failed to be removed during ERCP) are candidates for urgent surgical repair. Patients without any of these conditions were managed conservatively. Conservative management consisted of close monitorisation with physical examination, nasobiliary drainage, antibiotic administration and parenteral nutritional support. All patients were monitored with white blood cell count and C-reactive protein (daily). Surgery was planned immediately when there is hypotension (systolic blood pressure ≤90 mmHg), tachycardia (heart rate ≥120/min), fever (axillary temperature ≥38°C), worsening of abdominal symptoms and signs (signs of peritonitis).

## Results

Between March 2007 and March 2013, 2972 ERCPs were performed, 28 (0.94%) of which resulted in ERCP-related perforations. 10 of them were men (35.8%) and 18 women (64.2%). Mean age was 53.36±14.12 years with a range of 28 to 78 years. ERCP was performed for treatment of bile duct stones in 20 patients with additional cholangitis in 7 patients, 4 patients for cholangitis, for benign biliary stricture in 2 patients and for pancreas head cancer in 2 patients. A complete ERCP procedure includes cannulation, sphincterotomy, and basket-balloon instrumentation for stone removal or relieving the bile duct passage.

**Figure 1:**
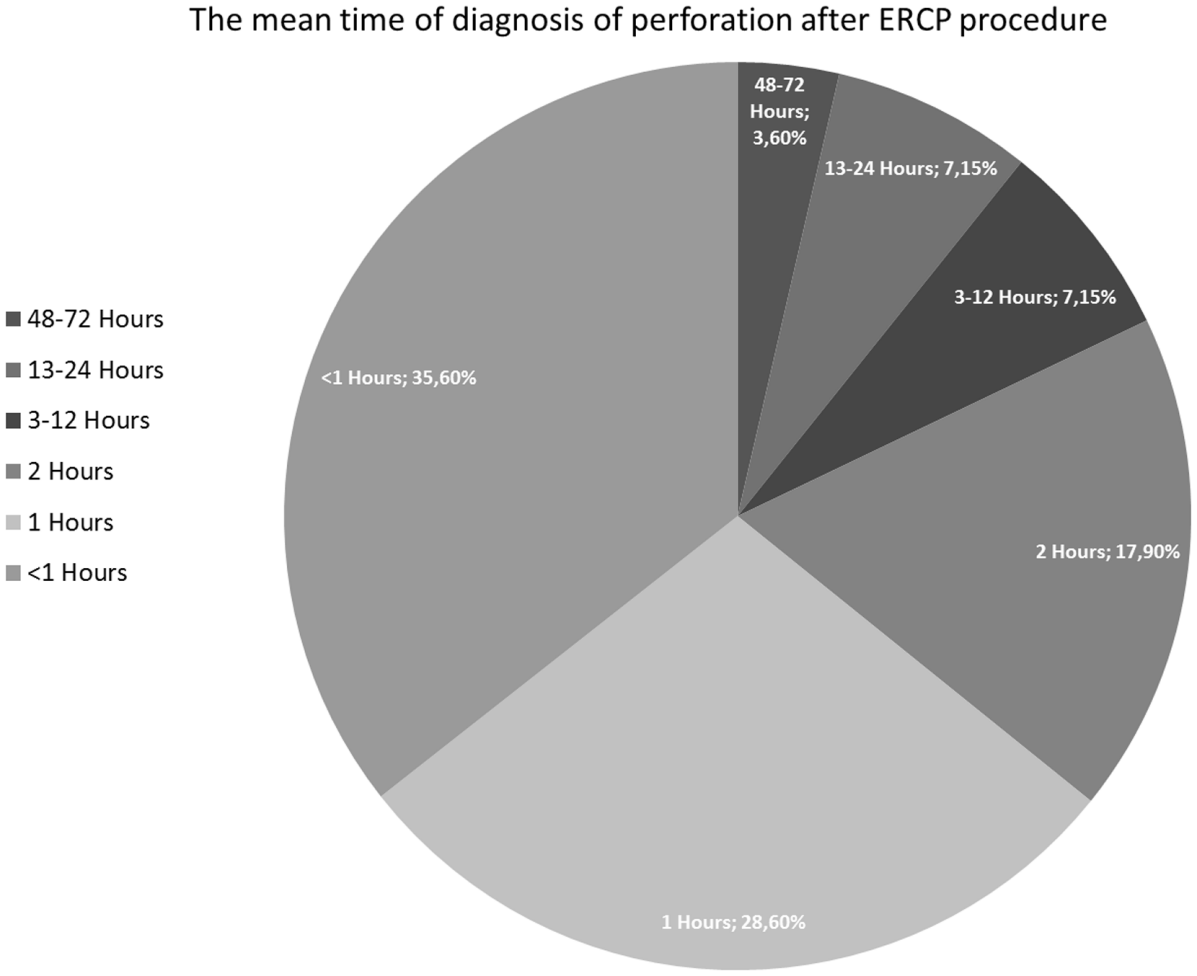
The figure shows the mean time of diagnosis after ERCP procedure (hours).

ERCP-related perforation during the intervention was suspected in 23 patients, only 10 (35.7%) of the perforations were diagnosed during ERCP whereas the remaining 18 were (64.3%) diagnosed by physical examination, trans-abdominal ultrasound, computerized tomography and abdominal radiography. Demonstration of a perforation during ERCP was accomplished by a limited contrast study through the endoscope. Severe post-procedural abdominal pain with/without pancreatitis, signs of peritonitis, fever and increased levels of CRP and white blood cells were accepted as suspected perforation. The mean time of diagnosis after ERCP procedure was 5.57 hours, ranged between 1 and 72 hours (**Figure 1**).

Conservative management was successful in 14 (50%) patients (**Table 2**), while 14 (50%) were managed surgically. In 6 patients, laparoscopic exploration was performed due to the failure of non-surgical management. Laparoscopic cholecyctectomy+Laparoscopic common bile duct exploration (LCBDE)+T-tube+drainage was performed in 5 of these patients. Although the remaining patient (63 year-old-male) underwent laparoscopic exploration on the 48th hour because of the progression of physical and laboratory findings, no sign of perforation (intra-and retroperitoneal fluid) was found. Laparoscopic cholecyctectomy+LCBDE+Stone extraction+Trans-cystic drain were successfully performed and the patient was discharged at the 12th post-operative day (**Table 3**). In 6 of the patients that ERCP-related perforation was suspected during or within 2 hours after ERCP, underwent to surgery primarily because of the extensive contrast extravasation on ERCP/CT and extraperitoneal or intraperitoneal fluid collection on CT (also retained biliary stone failed to be removed during ERCP). LCBDE+T-Tube+Drainage were performed; additionally laparoscopic cholecystectomy was added to the surgery in non-cholecystectomized three patients (**Table 4**). In the remaining two patients, although minimal invasive surgical approach was performed intra-abdominal abscess developed and required reoperation (**Table 5**). One of them was 63 year-old-female patient who underwent ERCP for CBD stone. The injury was near the ampulla consequent to precut sphincterotomy, showed contrast leakage. Laparoscopic cholecyctectomy+LCBDE+Stone extraction+T-Tube+intra-and retroperitoneal drainage were performed two hours after perforation. 72 hours after ERCP, signs of peritonitis, fever, and white blood cell counts were increased. That's why the surgical team decided to re-operate the patient. Intra-abdominal abscess was seen and pyloric exclusion+T-tube revision+gastrojejunostomi was performed. She was well discharged on the 14th day. The second patient was 68 years old female with the diagnosis of pancreas head cancer and liver metastasis underwent ERCP for biliary drainage. Type III injury in the distal common bile duct secondary to wire manipulation was suspected. LCBDE+T-Tube with intra- and retroperitoneal drainage was initially performed after two hours from ERCP. She was re-operated because of the intra-abdominal abscess on the 36th hour. There were two mortalities. The first one who was in failed non-surgical treatment group and died as a result of acute myocardial infarction on the 3rd day. Other patient with the diagnosis pancreas head cancer who underwent surgery for ERCP related perforation died because of sepsis at the 10th day. The mean length of hospitalization stay was 10.46±2.83 days. The overall mortality rate was 7.1%.

**Table 2:** Successful Nonsurgical Management of ERCP-Related Perforations.

Age	Gender	ERCP indication	Type of perforation	Time Between ERCP and diagnosis (hour)	Diagnosis of perforation	Radiologic Findings	LOS (day)	Outcome
51	F	CBD stones	II	0 h	ERCP	Minimal contrast extravasation	12 d	Survived
78	M	CBD stones	II	1 h	CT	Retroperitoneal air	11 d	Survived
69	F	Cholangitis	III	2 h	CT	Intra-and retroperitoneal air	9 d	Survived
54	F	CBD stones	II	1 h	CT, USG	Intra-and retroperitoneal air	14 d	Survived
68	M	Cholangitis	IV	1 h	CT	Retroperitoneal air	10 d	Survived
66	F	CBD Stones	III	0 h	ERCP	Minimal contrast extravasation	11 d	Survived
57	F	CBD stones	II	2 h	CT	Retroperitoneal air	11 d	Survived
49	F	Cholangitis	II	1 h	CT	Intra-and retroperitoneal air	9 d	Survived
28	F	CBD stones	II	0 h	ERCP	Contrast extravasation	12 d	Survived
34	F	CBD stones	III	2 h	CT	Retroperitoneal air	8 d	Survived
31	F	CBD Stones	II	0 h	CT	Intra-and retroperitoneal air	9 d	Survived
58	M	Benign biliary stricture	III	1 h	CT, USG	Intra-and retroperitoneal air, fluid collection	12 d	Survived
72	M	Pancreas Head Cancer	IV	0 h	CT	Retroperitoneal air	6 d	Survived
68	F	Benign biliary stricture	III	1 h	CT	Free air, fluid collection	11 d	Survived

***Footnotes:*** **ERCP:** Endoscopic Retrograde Cholangiopancreatography, **CT:** Computer Tomography, **USG:** Ultrasonography **LOS:** Length of Stay.

**Table 3:** Failed Non-Surgical Management of ERCP-Related Perforations.

Age	Gender	ERCP indication	Type of perforations	Time between ERCP and diagnosis (hour)	Time between ERCP and Operation (hour)	Type of Operation	LOS (day)	Outcome
54	F	Cholangitis+CBD stone	II	0 h	24 h	Laparoscopic cholecyctectomy+LCBDE+T-Tube+drainage	18 d	Survived
45	M	CBD stones	II	12 h	36 h	Laparoscopic cholecyctectomy+LCBDE+Stoneextraction+T-Tube+drainage	3 d	Ex (AMI)
54	F	Cholangitis+CBD stone	II	72 h	108 h	Laparoscopic cholecyctectomy+LCBDE+drainage	10 d	Survived
57	F	CBD stones	III	24 h	96 h	Laparoscopic cholecyctectomy+Stone extraction+LCBDE+T-Tube	11 d	Survived
71	F	CBD stones	II	18 h	48 h	Laparoscopic cholecyctectomy+LCBDE+T-Tube+drainage	12 d	Survived
63	M	Cholangitis+CBD stone	III	12 h	48 h	Laparoscopic cholecyctectomy+LCBDE+Trans-Cystic drain	12 d	Survived

***Footnotes:*** **ERCP:** Endoscopic Retrograde Cholangiopancreatography, **LOS:** Length of Stay, **AMI:** Acute Myocardia Infarcts, Ex:Exitus, **LCBDE:** Laparoscopic common bile duct exploration.

**Table 4:** Primary Minimal Invasive Surgical Management of ERCP-Related Perforations.

Age	Gender	ERCP indication	Type of Perforation	Time Between ERCP and diagnosis (hour)	Time between ERCP and Operation (hour)	Type of Operation	LOS (day)	Outcome
42	M	Cholangitis+CBD Stones	II	0 h	1 h	LCBDE+T-Tube+drainage	10 d	Survived
37	F	Cholangitis+CBD Stones	II	0 h	1 h	Laparoscopic cholecyctectomy+LCBDE+Trans-cytic drain+drainage	7 d	Survived
39	M	Cholangitis+CBD Stones	III	2 h	3 h	LCBDE+T-Tube	8 d	Survived
40	M	Cholangitis+CBD Stones	II	1 h	2 h	LCBDE+T-Tube+drainage	8 d	Survived
43	M	Cholangitis+CBD Stones	II	0 h	1 h	Laparoscopic cholecyctectomy+LCBDE+T-Tube+drainage	13 d	Survived
35	F	Cholangitis	II	2 h	3 h	Laparoscopic cholecyctectomy+LCBDE+T-Tube+drainage	4 d	Survived

***Footnotes:*** **ERCP:** Endoscopic Retrograde Cholangiopancreatography, **LOS:** Length of Stay, **LCBDE:** Laparoscopic common bile duct exploration.

**Table 5:** Failed primary minimal invasive surgical management of ERCP-related perforations.

Age	Gender	ERCP indication	Type of Perforation	Time Between ERCP and diagnosis (hour)	Time between ERCP and Operation (hour)	Type of Operation	LOS (day)	Outcome
63	F	CBD stones	II	0 h	1-) 2 h 2-) 72 h	Laparoscopic cholecyctectomy+LCBDE+Stone extraction+T-Tube+drainage Re-operation: Pyloric exclusion+T-Tube revision+gastrojejunostomy	14 d	Survived
68	F	Pancreas head cancer+liver metastasis	III	1 h	1-) 2 h 2-) 36 h	LCBDE+T-Tube+drainage Re-operation: explorative laparotomy+intra-abdominal abscess+drainage	10 d	Ex (Sepsis)

***Footnotes:*** **ERCP:** Endoscopic Retrograde Cholangiopancreatography, **LOS:** Length of Stay, **LCBDE:** Laparoscopic common bile duct exploration.

## Discussion

Although many patients with ERCP-related perforations can be managed expectantly, there is a dilemma for whom urgent operative intervention is necessary. In previous years some authors have suggested early operation for all endoscopic sphincterotomy perforations. However, with increasing experience with this rare but potentially lethal complication, there is increasing evidence that most perforations may be managed without surgery [20]–[23]. Early diagnosis of post-ERCP perforations is critical for successful management. Besides, the timing of operation is also important. The initial management is determined by the type and mechanism of injury. Progression of the symptoms and laboratory tests should be warning the surgeon for immediate surgical management. The key point is to decide who can be conservatively managed and who should be promptly operated.

Although several researches have classified ERCP-related perforations according to the location or mechanism of injury and have recommended various treatments, the most popular of these classifications was presented by Stapfer et al [11], [13], [14]. Stapfer et al classified perforations into four types according to the location and the mechanism of injury (**Table 1**) [11]. Type I perforations occur on the medial or lateral wall far from the ampulla and are caused by the endoscope itself or by the stent. Type II perforations are generally retroperitoneal, are classified as peri-vaterian, occur during sphincterotomy. Type III perforations are due to wire manipulation or basket instrumentation during stone retrieval and occur in the distal common bile duct. Type IV perforations are tiny retroperitoneal perforations caused by the use of compressed air during endoscopy. Another classification was suggested by Howard et al. includes three groups; group I: guidewire perforations, group II: periampullary perforations, group III: duodenal perforations [13]. Another frequently used classification was presented by Enns et al; group I: esophageal, gastric and duodenal perforations, group II: sphincterotomy-related perforations, group III: guidewire-related perforations [14]. There were 17 type-II perforations, 9 type-III perforations and 2 type-IV perforations in our case series according to the classification system of Stapfer et al. No type-I injury was observed.

The initial clinical presentation of patients with ERCP-related perforation is non-specific. The classic presentation of perforation, with severe epigastric pain, vomiting and epigastric tenderness progressing to generalized rigidity is only seen in the minority of cases. Moreover, the diagnosis is likely to be delayed if the patient has elevated amylase levels and the clinical presentation is attributed to post-ERCP pancreatitis. The most accurate diagnose can be made when the rupture is seen during the procedure. When there is a suspected duodenal perforation, an ultrasound or CT scan is a sensitive method to judge the existence of peritoneal, retroperitoneal emphysema or fluid collection [24], [25]. Genzlinger et al suggested that with routine post-ERCP computerized tomography in 13% to 33% of patients small amounts of retroperitoneal air may be detected, probably as a result of a post-procedural but non-significant micro-perforations [26]. Leukocytosis and fever that usually occur in the early phase are useful parameters for determining the management approach. Retroperitoneal nature of the injuries may mask severity; therefore, negative abdominal findings should not exclude surgery. Additionally, Mao et al suggested that subcutaneous emphysema is a sensitive physical sign that can be regarded as an effective parameter for an early diagnosis of perforation besides other radiologic examinations [16]. In our case series, perforation was suspected during the procedure in 23 (82%) patients and only in 8 (28.5%) of them it was diagnosed. Computerized tomography was performed within initial hours in 15 patients with suspected perforation to verify the diagnosis. These ratios are similar with other studies [3], [11], [13].

Most authors suggested to determine the type and mechanism of the perforation before selecting the optimal treatment method. Many studies reported that around 70% of patients with ERCP-related perforation could be managed conservatively [11], [17], [18]. Although, Stapfer et al reported that Type I injuries required prompt surgical interventions, recent studies recommended successful endoscopic treatments with endoscopic clippings, endo-loop applications and endoscopic closure devices [27]. Additionally, Stapfer suggested conservative treatment strategy for type II and III injuries (periampullary and bile duct injuries). In the presence of significant peritoneal findings, type II and III perforations should be treated by surgery. Furthermore, type IV (retroperitoneal air alone) perforations are not regarded as real perforations and should be treated conservatively. The rate of conservative management may vary depending on the management policies of the institutions. Our conservative treatment rate was 50% which is low compared to the other series. This is because these patients underwent surgery not only for the injury but also for the underlying disease, which could not be treated by ERCP.

The extent of surgery was proportional to the degree of injury, and the intraabdominal contamination. The basic principles of surgical therapy are repair of the leakage with diversion of the gastric contents and control for the source of the sepsis by means of external drainage [28]. Generally, surgical interventions that are used for the treatment of ERCP-related perforations are as follows; extensive drainage, repair with omental patch, pyloric exclusion, gastrojejunostomi, T-tube with or without cholecystectomy [16]–[18]. Sarli et al reported a wide range of operative procedures for the treatment of ERCP-related perforations, including simple retroperitoneal drainage, duodenal repair around a T-tube inserted into the perforation, common bile duct exploration+T-tube placement, duodenal diversion by antrectomy+gastrojejunostomy or gastrojejunostomy with pyloric exclusion and pancreaticoduodenectomy [29]. No article was found in the literature about the laparoscopic management of ERCP-related perforations. In a study about the comparison of LCBDE and ERCP for the treatment of common bile duct stones, it was pointed that in experienced hands LCBDE is a safe and feasible option with the advantages of minimal access [30].

**Figure 2:**
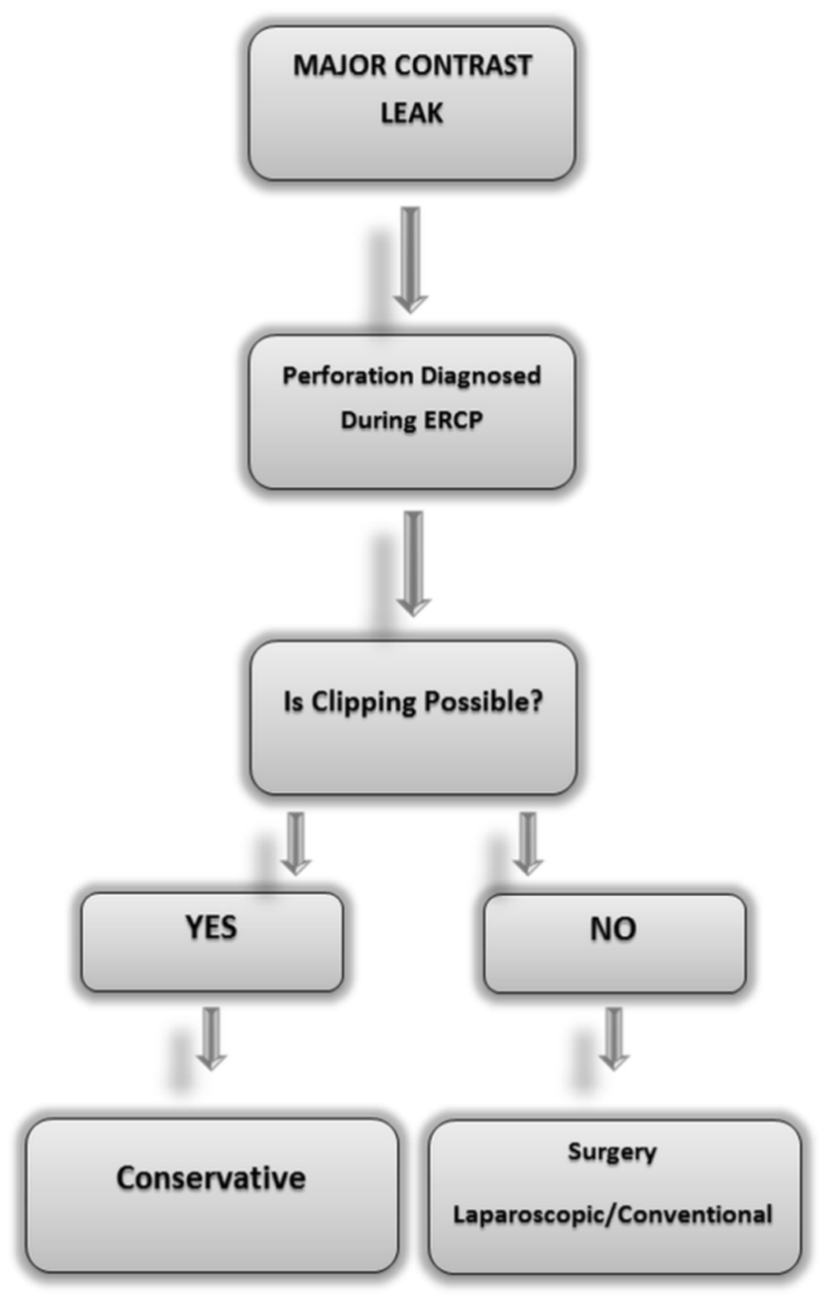
A simple management algorithm for the ERCP-related perforations.

To summarize, we performed LC+LCBDE+T-tube+intra-or/and retroperitoneal drainage in 6 patients due to the failure of non-surgical management and LCBDE+with or without LC+T-tube+intra-or/and retroperitoneal drainage for 8 patients as a primary management. This approach failed in 2 (14%) patients, and our surgical mortality rate was 7.1%. These rates were similar to literature (**Table 6**). In this technique we can solve the injury and also the underlying disease (extraction of bile duct stones) in the same intervention. The most important limitation of our study was lack of type I injuries. It should be kept in mind that minimally invasive management (endoclipping) could be attempted if the perforation is diagnosed during ERCP. We suggest that, in patients with cholelitiasis and choledocholithiasis if type II and III perforation occurs during ERCP, CBDE+LC+T-tube+drainage with nasogastric suction can be performed as primary treatment or when conservative treatment fails. Based on our findings, we propose a simple management algorithm which can be readily and easily used (**Figure 2**). To design a prospective study about laparoscopic approach is not possible due to major ethical issues. That's why the evaluation of this approach can only be made by retrospective case series. The source of severe sepsis and peritonitis may not be revealed objectively by laparoscopy laparoscopy which is a serious problem that needs to be resolved.

**Table 6:** Reported perforation rates with ERCP.

Study	Length of Study	Number of ERCP's	Perforations	Operations	Mortality	Year
***Chaudhary and Aranya *** [*31*]	10 years	750	10(1.3)	10(100%)	2(20%)	1996
***Loperfido et al *** [*6*]	2 years	3356	28(0.83%)	10(35.7%)	4(14.3%)	1998
***Stapfer et al *** [*11*]	5 years	1413	14(0.99%)	9(64.3%)	2(14.3%)	2000
***Preetha et al *** [*28*]	9 years	4030	18(0.45%)	18(100%)	3(16.7%)	2003
***Christensen et al *** [*32*]	2 years	1177	13(1.1%)	2(15.4%)	1(7.7%)	2004
***Wu et al *** [*33*]	6 years	6620	30(0.45%)	10(33.3%)	5(16.7%)	2006
***Fatima et al *** [*18*]	11 years	12427	76(0.6%)	22(28.9%)	5(6.6%)	2007
***Cotton et al *** [*4*]	12 years	11497	16(0.14%)	11(68.8%)	1(6.3%)	2009
***Morgan et al *** [*34*]	13 years	12817	24(0.2%)	10(41.7%)	1(4.2%)	2009
***Gurung et al *** [*35*]	2 years	423	1(0.2%)	1(100%)	0 (0%)	2014
***Katsinelos et al *** [*36*]	7 years	2837	3(0.11%)	1(0.035)	0 (0%)	2014
***Present Series***	6 years	2972	28(0.94%)	20(71.4%)	2 (7.1%)	2014

***Footnotes:*** **ERCP:** Endoscopic Retrograde Cholangiopancreatography.

## Conclusions

Successful management of ERCP-related perforation requires immediate diagnosis and early decision to decide whether to manage conservatively or surgically. While patients with type I perforation would invariably require immediate surgical intervention, those with type II or III, IV may often be managed conservatively. Otherwise, these types of injuries with retained stones and unrelieved bile obstruction should be explored. Although traditionally conventional surgical approaches have been suggested for the treatment of perforations, laparoscopic techniques may be used in well-chosen cases especially in type II, III and IV perforations.
